# Increased serum hepcidin-25 level and increased tumor expression of hepcidin mRNA are associated with metastasis of renal cell carcinoma

**DOI:** 10.1186/1471-2407-9-270

**Published:** 2009-08-05

**Authors:** Takao Kamai, Naohisa Tomosugi, Hideyuki Abe, Kyoko Arai, Ken-Ichiro Yoshida

**Affiliations:** 1 Department of Urology, Dokkyo Medical University, Tochigi, Japan; 2 Proteomics Research Unit, Division of Advanced Medicine, Medical Research Institute, Kanazawa Medical University, Ishikawa, Japan

## Abstract

**Background:**

Hepcidin has an important role in iron metabolism. We investigated whether hepcidin was involved in renal cell carcinoma (RCC).

**Methods:**

We measured serum hepcidin-25 levels in 32 patients by liquid chromatograpy (LC)-mass spectrometry (MS)/MS, and assessed hepcidin mRNA expression in paired tumor and non-tumor tissue samples from the surgical specimens of 53 consecutive patients with RCC by real-time reverse transcription polymerase chain reaction.

**Results:**

The serum hepcidin-25 level was higher in patients with metastatic RCC than nonmetastatic RCC (*P *< 0.0001), and was positively correlated with the serum interleukin-6 and C-reactive protein levels (*P *< 0.001). Expression of hepcidin mRNA was lower in tumor tissues than in non-tumor tissues (*P *< 0.0001). The serum hepcidin-25 level was not correlated with the expression of hepcidin mRNA in the corresponding tumor tissue specimens from 32 patients. Hepcidin mRNA expression in tumor tissue was correlated with metastatic potential, but not with histological differentiation or tumor stage. Kaplan-Meier analysis showed that over expression of hepcidin mRNA was related to shorter overall survival in RCC patients. Univariate analysis (Cox proportional hazards model) showed that the hepcidin mRNA level was an independent prognostic factor for overall survival.

**Conclusion:**

Our findings suggest that a high serum hepcidin-25 level may indicate the progression of RCC, and that upregulation of hepcidin mRNA expression in tumor tissue may be related to increased metastatic potential.

## Background

Renal cell carcinoma (RCC) is the most common malignant tumor of the kidneys and the third most common malignancy in the urological field. More than 50% of all RCCs are found incidentally, which results in a high percentage of patients with metastasis at the time of diagnosis [1]. In patients with disseminated RCC, conventional chemotherapy, radiotherapy, and immunotherapy have all been tried with limited efficacy [1-4]. Thus, more effective therapy is urgently needed to improve the outcome for patients with advanced RCC. Better understanding of the biology and genetics of RCC have shed light on possible targeted approaches to metastatic disease [5]. The pathway involving the Hipple-Lindau (VHL) tumor suppressor gene, hypoxia-inducible factor-1 (HIF-1) alpha, and vascular endothelial growth factor (VEGF) is important for the growth of clear cell RCC, and targeting this pathway is a novel approach to the treatment of metastatic RCC.

Iron is required as a cofactor for the catalytic activity of HIF prolyl hydroxylases (HPHs) [6]. Iron is a pivotal nutrient for cell growth and cell cycle regulation, so iron chelators may be useful for the treatment of cancer [7]. It has been reported that iron is more abundant in RCCs than in the surrounding non-tumor tissues [8]. Therefore, it is of interest to investigate the factors that modulate iron metabolism in RCC. Hepcidin is a pivotal regulator of iron metabolism because it controls the efflux of iron from enterocytes, hepatocytes, and macrophages by internalization and degradation of the iron exporter (ferroportin), and also regulates the plasma iron level [9,10]. Therefore, the role of hepcidin in human cancer deserves to be studied. While there have been a few reports about the role of hepcidin in some cancers [11,12], little is known about its influence on RCC. In the present study, we examined serum hepcidin-25 levels by liquid chromatograpy (LC)-mass spectrometry (MS)/MS and compared hepcidin mRNA expression between RCC tissues and non-neoplastic tissues from the same resected specimens by real-time reverse transcription-polymerase chain reaction (RT-PCR). The relationship between the serum level of hepcidin-25 and hepcidin mRNA expression or the clinicopathologic features of RCC patients was also examined. Furthermore, we assessed whether hepcidin could be used to predict the prognosis of RCC patients. Such information might lead to with regard to the role of hepcidin in RCC.

## Methods

### Patients and tissue specimens

We studied 53 consecutive Japanese patients (37 men and 16 women) aged 35 to 77 years (mean age: 62.9 years), who were diagnosed as having clear cell RCC from 2002 to 2007. All patients routinely underwent imaging studies (CT and/or MRI) for preoperative staging prior to radical nephrectomy. The postoperative follow-up period ranged from 2 to 84 months (median: 29 months). Patients underwent surgery before receiving any other therapy.

In every patient, three different tumor sites and various parts of the non-neoplastic kidney were harvested for this study. The resected tissues were stored at -80°C, as described previously [13-15]. The tumor grade and clinical stage were determined according to the Fuhrman grading system and the TNM classification, respectively [16,17]. This study was conducted in accordance with the Declaration of Helsinki and institutional review board approval was obtained. Each patient signed a consent form that had been approved by the Committee on Human Rights in Research of our institution.

Postoperative immunotherapy with IFN-α was usually administered to patients with extra-renal involvement. These patients received 3, 5, or 6 million units of natural human IFN-α intravenously or intramuscularly two or three times a week for 12 weeks to 6 months, or until tumor progression occurred. The dose of IFN-α was decreased if grade 3/4 toxicity occurred.

### Measurement of serum hepcidin-25

The serum level of hepcidin-25 was measured in preoperative blood samples obtained from 32 patients by LC-MS/MS at Medical Care Proteomics Biotechnology Co., Ltd. (Ishikawa, Japan), as described previously [18]. We compared the serum hepcidin-25 level with hepcidin mRNA expression in the 32 corresponding tumors, and also with the serum levels of interleukin (IL)-6 (mean = 1.77 pg/ml, n = 32, range: 0.447–9.96 pg/ml, R&D System Co. Ltd.) and C-reactive protein (CRP) (normal < 0.3 mg/dl) in the same 32 patients.

### Real-time RT-PCR assay

Total RNA was purified from all 53 sets of tumor and non-tumor tissue samples with an RNA preparation kit ("High Pure RNA Kit"; Roche Diagnostic Ltd., Germany), and was used as the template for cDNA synthesis. A 100 μL reaction mixture containing 1 mg of random hexamers and 100 units of MMLV-reverse transcriptase was incubated at 25°C for 10 min, at 42°C for 30 min, and then at 99°C for 5 min. The hepcidin gene expression profile was analyzed with an ABI PRISM 7700 Sequence Detection System (Applied Biosystems, Foster City, CA) using the SYBR Green method. The following primers were employed to amplify the indicated genes in the primary tumors after confirming their specificity: hepcidin, sense; 5'-TTCCCCATCTGCATTTTCTG-3', anti-sense; 5'-TCTACGTCTTGCAGCACATCC-3'; b_2_-microglobulin, sense; 5'-ACCCCCACTGAAAAAGATGA-3', anti-sense; 5'-ATCTTCAAACCTCCATGATG. Real-time RT-PCR was performed in a 25 μL reaction mixture containing 20 ng of sample cDNA, 100 nM sense primer, 100 nM anti-sense primer, and 12.5 μL of SYBR Green PCR Master Mix (Applied Biosystems). PCR was carried out by performing 45 cycles of hearting at 95°C for 15 sec and 60°C for 1 min. A standard curve for each mRNA was generated by using five-fold dilutions of a control RNA sample (25×, 5×, 1×, 0.2×, 0.04×). The level of expression of the target gene mRNA was calculated as a ratio to that of b_2_-microglobulin [14,15]. The mean value obtained by real-time RT-PCR of three tissue samples was used for analysis, as described previously [14,15].

### Statistical analysis

The results of LC-MS/MS and real-time RT-PCR were analyzed by using the Mann-Whitney *U *test to compare two groups and the Kruskal-Wallis test to compare three groups, as described previously [13-15]. Hepcidin mRNA expression, tumor grade, and tumor stage were assessed for their impact on survival by Cox proportional hazards analysis using univariate and multivariate models. The Kaplan-Meier method was employed to estimate survival, and differences were assessed by the log-rank test. A *P *value of less than 0.05 was considered significant. Data were analyzed with commercially available software.

## Results

### Serum hepcidin-25 level and tumor characteristics

**Figure 1:**
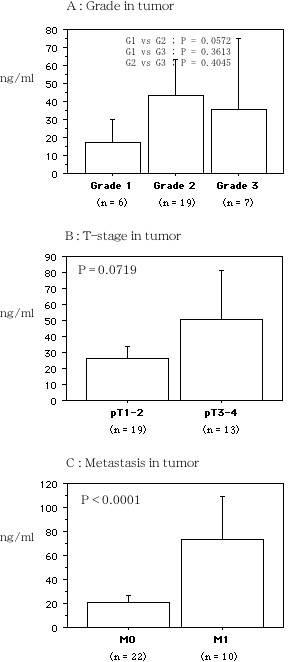
**Serum hepcidin-25 concentration in the patients with renal cancers**. A; In Grade 1 to 3 tumors. B; In pT1–2 and pT3–4 tumors. C: In metastasis (+ : 1) and (- : 0). The data show the 95% confidential interval.

**Figure 2:**
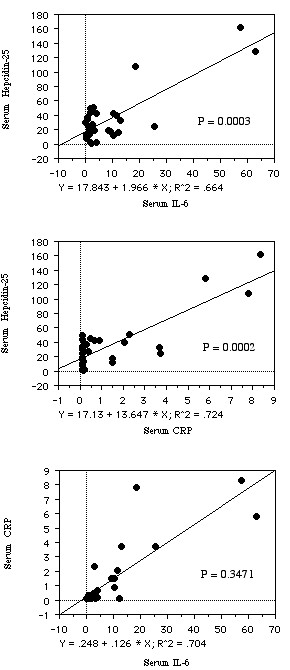
**Spearman rank correlation coefficient relationship between serum concentrations of hepcidin-25, IL-6, and CRP**.

There was no relationship between the serum hepcidin-25 level and the histological grade of RCC (mean ± S.D., grade 1, 17.02 ± 10.32; grade 2, 43.37 ± 40.04; grade 3, 35.58 ± 37.78, *P *= 0.1492, Figure 1A), or the tumor stage (pT1–2, 26.21 ± 14.58; pT3–4, 50.75 ± 50.04, *P *= 0.0719, Figure 1B). However, serum hepcidin-25 was associated with metastasis (M0, 21.05 ± 12.42; M1, 73.14 ± 47.13, *P *< 0.0001, Figure 1C). The serum IL-6 level was also correlated with metastasis (M1, 19.41 ± 23.79; M0, 4.62 ± 6.19, *P *= 0.0095), and it increased along with a higher tumor grade (grade 1, 3.82 ± 2.21; grade 2, 9.74 ± 4.41; grade 3, 12.08 ± 3.57, *P *= 0.0836) and higher stage (pT1–2, 4.88 ± 6.63; pT3–4, 14.54 ± 20.99, *P *= 0.0926). A higher serum CRP level was associated with poorly differentiated RCC (grade 1, 0.10 ± 0.01; grade 2, 1.32 ± 2.33; grade 3, 2.56 ± 2.59, *P *= 0.0036), local invasion (pT1–2, 0.43 ± 0.90; pT3–4, 2.52 ± 2.86, *P *= 0.0014), and metastatic disease (M1, 3.45 ± 3.15; M0, 0.52 ± 0.92, *P *= 0.0004). The serum hepcidin-25 level was positively correlated with the serum levels of IL-6 and CRP (r^2 ^= 0.664, *P *= 0.0003, and r^2 ^= 0.724, *P *= 0.0002, respectively, Figure 2A,B). There was no correlation between IL-6 and CRP levels (r^2 ^= 0.704, *P *= 0.3471, Figure 2C). The serum hepcidin-25 level was not correlated with the expression of hepcidin mRNA in the corresponding tumor tissue samples from 32 patients (data not shown).

### Hepcidin mRNA expression and pathologic characteristics

**Figure 3:**
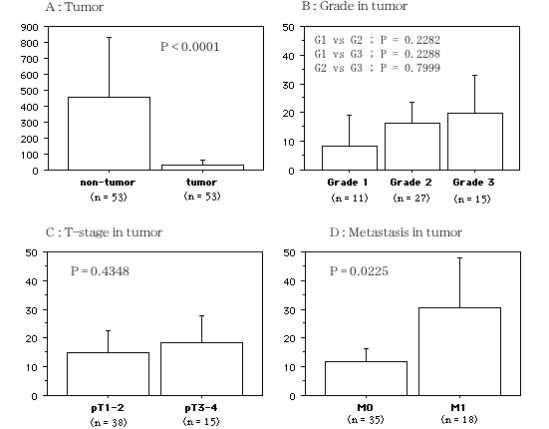
**Hepcidin mRNAs in tumors**. A; In tumor vs. non-tumor tissues. B; Grade 1 to 3 tumors. C; In pT1–2 and pT3–4 tumors. C: In metastasis (+ : 1) and (- : 0). The data show the 95% confidential interval.

Hepcidin mRNA was detected in both the tumors and in non-tumorous kidney tissues, (mean ± S.D. 30.77 ± 100.51 and 456.83 ± 1280.80, respectively, *P *< 0.0001, Figure 3A). The level of hepcidin mRNA expression in RCC was not related to the tumor grade (mean ± S.D. grade 1, 8.34 ± 8.66; grade 2, 16.36 ± 18.96; grade 3, 19.79 ± 23.12, *P *= 0.4249, Figure 3B) or the tumor stage (pT1–2, 14.88 ± 19.15; pT3–4, 18.56 ± 20.13, *P *= 0.4348, Figure 3C). However, hepcidin mRNA expression was higher in the patients with metastasis (M1) compared to those with tumors confined to the kidney (M0) (11.76 ± 13.23 vs. 30.44 ± 27.58, *P *= 0.0225; Figure 3D).

### Hepcidin mRNA expression and survival

**Figure 4:**
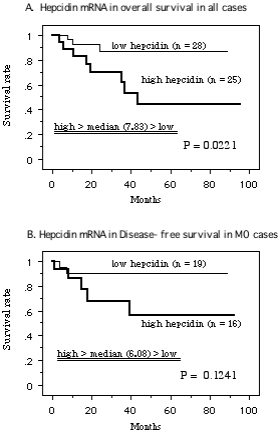
**Survival curve based on the median values of mRNA expression of hepcidin in tumors, the cases were divided into two groups at this levels – high and low expression**.

The median level of hepcidin mRNA expression in tumor tissue was 7.83. Patients were divided into two groups (high and low hepcidin expression) based upon whether their hepcidin mRNA level was above or below this median value. Kaplan-Meier analysis of patients with low or high hepcidin mRNA expression suggested that higher expression by RCC was significantly related to shorter overall survival (*P *= 0.0221; Figure 4A). Univariate analysis according to the Cox proportional hazards model showed that overall survival was significantly influenced by Karnofsky performance status, tumor grade, stage, metastasis, and hepcidin mRNA expression (Table 1). According to multivariate analysis, the performance status and the tumor grade were independent prognostic factors (Table 1). Patients without distant metastasis at the time of nephrectomy (M0; n = 35) were also divided into groups with hepcidin mRNA levels above or below the median value of 6.08. In these patients with localized tumors, Kaplan-Meier analysis showed that higher hepcidin mRNA expression had no influence on disease-free survival (Figure 4B).

**Table 1:** Cox regression analysis for various potential prognostic factors in survival

			Overall survival in 53 clear cell renal cell carcinomas			
Variable	Unfavorable/favorable characteristics	No. of Patients	Analysis	Relative risk	95% confidential interval	P value

			Univariate (U)	18.529	5.287 – 27.819	< 0.0001
Karnofsky PS	< 80%/> 80%	5/48				
			Multivariate (M)	24.718	2.793 – 11.729	0.0029
						

						
			U	35.194	4.359 – 284.127	0.0008
Grade	4/3/2/1	0/15/30/8				
			M	85.943	4.123 – 1791.603	0.004
						

						
			U	10.667	1.349 – 84.339	0.0248
pT	4.3/2.1	15/38				
			M	6.512	0.448 – 94.642	0.17
						

						
			U	5.585	1.558 – 20.017	0.0083
Metastasis	M1/M0	18/35				
			M	3.188	0.622 – 11.612	0.1273
						

						
			U	4.177	1.105 – 15.784	0.0351
Hepcidin mRNA	high/low	25/28				
			M	5.824	0.846 – 40.092	0.0734

## Discussion

There were three main findings of this study. First, the serum hepcidin-25 level was higher in patients with metaststic RCC than in those without metastatic tumors. Second, expression of hepcidin mRNA was increased in metastatic RCCs compared with non-metastatic RCCs. Third, although the follow-up period was too short to draw definite conclusions about the relationship between survival and hepcidin mRNA expression, higher tumor expression of hepacidin mRNA was related to unfavorable overall survival in our RCC patients.

Hepcidin is upregulated in response to an increase of body iron stores or the onset of infection and is downregulated by anemia or hypoxia, while it is also an acute phase reactant induced by inflammation that shows antimicrobial activity [9,10]. It has been reported that induction of hepcidin is stimulated by IL-6 [19]. The circulating level of CRP is associated with the stage and outcome of RCC [20-23]. Although our data showed no positive correlation between serum IL-6 and CRP levels, elevation of the CRP level is primarily determined by an increase of circulating IL-6 [24], and the IL-6 level is correlated with the serum CRP level as well as with tumor histological grade and metastasis [25]. It has been reported that IL-6 is produced by most RCCs and it is assumed to be an autocrine growth factor [26,27]. These findings suggest that the serum IL-6 and CRP levels are associated with tumor aggressiveness, so that elevation of IL-6 and CRP might reflect the poor general condition of a patient. Motzer et al. [28] identified five prognostic factors (Karnofsky performance status, time from diagnosis of RCC to treatment, serum lactate dehydrogenese, corrected serum calcium, and hemoglobin) that were correlated with overall survival in patients who had metastatic RCC and were receiving systemic therapy, and this is known as the Memorial Sloan-Kettering Cancer Center (MSKCC) classification. The prognosis of our patients was consistent with the MSKCC model. In the present study, patients from the unfavorable risk group according to the MSKCC classification had higher serum levels of IL-6, CRP, and hepcidin-25 (data not shown). Recently, Ganz et al. reported that serum hepcidin levels were increased in patients who had inflammation and an elevated serum CRP level, multiple myeloma, or chronic kidney disease, indicating that hepcidin is influenced by physiologic, pathologic, and genetic factors, and that it provides useful information about the etiology of iron disorders [29]. At present, it remains unclear how elevation of serum hepcidin-25 reflects and/or is involved in the progression of RCC, but our observations showed that the serum hepcdin-25 level was correlated with the levels of IL-6 and CRP and that elevation of all three factors was associated with metastasis of RCC. Since there was no relationship between the serum hepcidin-25 level and tumor expression of hepcidin mRNA in this study, the origin of circulating hepcidin-25 might not be the cancer cells. Production of hepcidin-25 is induced in the liver by IL-6 [19]. Kulaksiz et al. performed immunohistochemistry of human, mouse, and rat kidneys, which revealed similar patterns of hepcidin immunoreactivity in the 3 species, including strong expression in the cortical part of the thick ascending limb of the loop of Henle and the collecting tubules, moderate expression in the medullary part of the thick ascending limb and collecting ducts, as well as in collecting ducts of the papilla, and no expression in the proximal convoluted tubules and the descending and ascending thin limbs [30]. Clear cell RCC is thought to arise primarily from the proximal convoluted tubules [31]. Therefore, our observation of lower hepcidin mRNA expression in tumors tissues compared with normal kidney tissue may reflect this origin.

Metastasis is the most common fatal complication of cancer and it involves dissemination of tumor cells to organs distant from the primary lesion, so transport of tumor cells through the body fluids is required [32]. The present study showed that increased hepcidin mRNA expression by RCC was associated with metastasis and survival in patients. The meaning of local hepcidin expression remains unclear. However our findings imply the alteration to metastatic state with motility from steady state for proliferation. Cell characteristics in cancer affected during Epithelial-mesenchymal transition resulting in altered cell-cell and cell-matrix interactions, cell motility and invasiveness [33]. Iron metabolism may be different in the state of migration from proliferation and it may affect during hepcidin expression. Moreover, angiogenesis is required for tumor metastasis and progression, and thus represents a rational target for therapeutic intervention [34]. HIF-1 is a key regulator of tumor angiogenesis [35]. After oncogenic transformation, HIF-1 protein is stabilized and translocated into the nucleus where it regulates the expression of various genes, including the key angiogenic growth factor VEGF. In the majority of sporadic clear cell RCCs, inactivation of the *VHL *tumor suppressor gene is observed, and genetic or epigenetic silencing of this gene may induce overexpression of HIF-1 [6]. Because clear cell RCC is a vascular tumor that shows upregulation of VEGF expression and metastasizes early via the hematogenous route, its prognosis is unfavorable [36]. Therefore, HIF-1 is an attractive target for the development of new anticancer therapies [37]. Iron is required as a cofactor for the catalytic activity of HPHs [6], and is also a pivotal nutrient for cell growth and cell cycle regulation [7]. Furthermore, it has been reported that iron is more abundant in RCC tissues than in non-tumor tissues [8]. Although we did not examine tumor and non-tumor tissue iron levels, hepcidin mRNA expression was higher in the RCCs with metastasis than in those without metastasis, indicating that an increase of hepcidin mRNA expression might be associated with elevation of the iron content in tumor cells.

While the origin of hepcidin-25 and hepcidin mRNA in hepatocytes remains elusive, expression of hepcidin mRNA in surgical specimens of hepatocellular carcinoma (HCC) was lower than in non-tumorous liver tissue and was not correlated with the serum hepcidin-25 level, as was also found in the current study of RCC [11]. Moreover, hepcidin mRNA expression was not related to the histological grade, vascular invasion, or recurrence of HCC [11]. Hepcidin mRNA expression was detected in 10 out of 34 surgical specimens from patients with colorectal cancer, but was not detected in the majority of matched non-tumorous tissues [12]. It has been reported that an increase of colonocyte iron levels can lead to upregulation of Wnt signalling, which has been shown to have a crucial role in colorectal carcinogenesis [38,39], indicating that hepcidin might have a pro-oncogenic role by internalization and degradation of the cellular iron export protein ferroportin. Taken together, these findings suggest that hepcidin might have differing roles in different cancers, but an increase of iron based on increased hepcidin mRNA expression in tumor cells might be necessary for the metastasis of RCC. In the future, we should examine whether hepcidin is involved in the VHL-HIF1-VEGF signaling pathway and in the internalization and degradation of ferroportin in RCC.

As described above, hepcidin regulates the plasma iron level by internalization and degradation of ferroportin in enterocytes, hepatocytes, and macrophages [9,10]. In addition, the hepcidin level increases in response to infection and it is an acute phase reactant induced by inflammation that shows antimicrobial activity [9,10]. Therefore, an increase of the serum hepcidin-25 level in patients with metastatic RCC might prevent tumor cells from utilizing iron by upregulating tumor cell hepcidin as an anticancer mechanism, as well as showing antimicrobial activity if infection occures. It is likely that hepcidin plays multiple roles; e.g., hepcidin mRNA acts locally within tumors and promotes their metastatic poteintial, while hepcidin-25 also acts systemically to alleviate the tumor burden in response to the progression of RCC.

Our study included a relatively small number of patients and the follow-up period was too short to draw definite conclusions regarding the possible relationship between the serum hepcidin-25 level or tumor hepcidin mRNA expression and the prognosis of RCC. However, our findings suggested that serum hepcidin-25 may be an indicator of metastasis and that an increase of hepcidin mRNA expression in RCC may be associated with metastatic potential. Accordingly, serum hepcidin-25 may be a useful prognostic indicator and there is a potential role of hepcidin in the metastasis of RCC. In the future, we should study hepcidin protein expression to strengthen our data about its role and carcinogenesis in metastasis. We should also define the downstream targets of hepcidin and study the expression or activity of such targets using cultured tumor cell lines or tissue samples.

## Conclusion

This study showed that an elevated serum hepcidin-25 level is indicative of metastatic disease, while over expression of hepcidin mRNA in tumor tissue is associated with the metastatic potential of RCC.

## Competing interests

The authors declare that they have no competing interests.

## Authors' contributions

TK and NT initiated the study, participated in its design and coordination, carried out the study, performed the statistical analysis, and drafted the manuscript. HA and KA carried out the study. K-IY participated in the design of the study and helped to draft the manuscript. All authors read and approved the final manuscript.

## Pre-publication history

The pre-publication history for this paper can be accessed here:

http://www.biomedcentral.com/1471-2407/9/270/prepub
